# New Approaches to Optimize Somatic Embryogenesis in Maritime Pine

**DOI:** 10.3389/fpls.2019.00138

**Published:** 2019-02-19

**Authors:** Isabel Arrillaga, Marian Morcillo, Israel Zanón, Francisco Lario, Juan Segura, Ester Sales

**Affiliations:** ^1^ERI BiotecMed, Departamento de Biología Vegetal, Facultad de Farmacia, Universidad de Valencia, Valencia, Spain; ^2^TRAGSA, Vivero de Maceda, Carretera Maceda – Baldrei, Ourense, Spain; ^3^Departamento de Ciencias Agrarias y del Medio Natural, Escuela Politécnica Superior, Instituto Universitario de Ciencias Ambientales, Universidad de Zaragoza, Huesca, Spain

**Keywords:** embryo maturation, gene expression, hormone content, *LEC1*, maternal effect, temperature, water availability, *WOX2*

## Abstract

Maritime pine (*Pinus pinaster* Aiton) is a coniferous native of the Mediterranean basin. Because of its adaptability to a wide range of environmental conditions, the species have become a model for studies in coniferous forest management and functional genomics. Somatic embryogenesis (SE) has been so far, the preferred biotechnological strategy for maritime pine breeding programs initiated at the middle-end of the 20th century. To overcome the limitations of the induction and maturation phases in maritime pine SE, we analyzed the possible maternal influence on the embryogenic capability of megagametophytes from controlled crosses, as well as the effect of the temperature and water availability during SE process on the production of plants. A strong maternal effect on the embryogenic potential of maritime pine megagametophytes was observed in our experiments using half-sib and full-sib progenies, while paternal effect was almost undetectable. Besides, it seems possible to improve somatic embryo production of maritime pine megagametophytes by adjusting optimal temperature throughout the process: 28°C during induction and proliferation, and 23°C during the maturation phase. Using induction and proliferation media with reduced water availability (6 g/L Gelrite) can also increase embryo production. Since other limitation of maritime pine SE is culture decline of embryogenic masses (EMs), that reduces embryo yield and germination, we assessed the profile of ABA and IAA and the expression of two embryogenesis-related genes (*LEC1* and *WOX2*) during maturation of EMs of two morphotypes that differed in their maturation capability. Spiky morphotype (SK), with high maturation capability, had a steady increase in both hormones along the 12 weeks of the maturation, whereas ABA content in smooth morphotype picked at the 4th week and dropped. EMs with this morphotype also had a higher IAA content at the beginning of the maturation. A decrease of *LEC1* and *WOX2* gene expression over the course of embryo development was found to be characteristic of the SK with high maturation capability.

## Introduction

*Pinus pinaster* Aiton is one of the most commercial and economically valuable tree species in the Mediterranean Basin. Due to its capacity to live in a wide range of ecological conditions, it has been traditionally used in breeding and reforestation programs, and as a model organism for the study of stress responses in conifers (Arrillaga et al., 2014). In breeding programs, clonal multiplication of selected families from controlled crosses is a powerful method of capturing maximal genetic gain (Niskanen et al., 2004), therefore protocols for micropropagation of maritime pine through axillary bud breaking, adventitious bud differentiation and SE that have been reported (Cano et al., 2018 and references therein) constitute a particularly useful tool. Among them, SE is the most efficient biotechnological approach for conifer clonal propagation, and then a powerful strategy for research and breeding (Klimaszewska et al., 2016; Lelu-Walter et al., 2016).

Because SE in pines and in other conifers is a multi-stage process, each stage requires adjustments to maximize its efficiency. Previous work developed for the species demonstrated higher initiation rates of SE from immature megagametophytes containing zygotic embryos at the precotyledonary stage (Humánez et al., 2012). However, the initiation frequency of SE also depended on the genotype, making the technique still insufficient for commercial application. Furthermore, the improvement in SE initiation is important for developing varietal lines, as well as to managing genetic diversity in the breeding population (Park et al., 2006). Experiments designed to establish the extent of genetic control in SE initiation have been conducted in a few pine species suggesting a stronger maternal than paternal effect on culture initiation (Niskanen et al., 2004; MacKay et al., 2006; Lelu-Walter et al., 2008).

Embryogenic cultures of gymnosperms proliferate as EMs that typically have a whitish to translucent appearance and may have a granular to spiky (SK) morphotype due to early embryos protruding at their surface (see Gautier et al., 2018 and references therein). Modifications in water availability, either by solute-imposed water stress or by physical restriction, will impact the development of embryogenic tissue (Klimaszewska et al., 2000). In this respect, the effect of different concentrations of gellan gum on maritime pine somatic embryo maturation (Morel et al., 2014a) and the combination of different temperatures and water availability during the initial stages of SE in *P. radiata* (García-Mendiguren et al., 2015) and *P. halepensis* (Pereira et al., 2016) have been reported.

Although ABA-supplemented medium is required for conifer somatic embryo maturation, a certain number of somatic embryogenic lines in a given species fail to produce mature somatic embryos even in the presence of optimized concentrations of this hormone. In different *Picea glauca* x *engelmanni* genotypes the ability of embryogenic tissue to utilize ABA from the medium may reflect their maturation capability (Kong and von Aderkas, 2007). The switch from EMs proliferation to somatic embryo development and maturation has recently been investigated by both transcriptome and proteome profiling (Morel et al., 2014a; Plomion et al., 2016; Trontin et al., 2016; Klubicová et al., 2017; Gautier et al., 2018). In maritime pine, yield and quality of mature somatic embryos is strongly dependent on the embryogenic line (Lelu-Walter et al., 2016; Cano et al., 2018). Because of this, morphological criteria are usually applied to select desirable EMs for maturation. Thus, SK, characterized by high amounts of protruding immature SE at the periphery, are associated with a high maturation potential, whereas in smooth lines (SM) immature embryos are scarcely visible, and fail to produce mature somatic embryos (Ramarosandratana et al., 2001; Breton et al., 2006). Nevertheless, hormonal and molecular differences between SK and SM morphotypes have not been yet reported.

To date, knowledge on the molecular factors initiating and controlling SE are scarce (Uddenberg et al., 2011; Lara-Chavez et al., 2012; Morel et al., 2014a; Rupps et al., 2016; Gautier et al., 2018). However, studies on some of the genes associated with conifer embryogenesis as *LEAFY COTYLEDON* (*LEC1*), *WUSCHEL-related HOMEOBOX2* (*WOX2*) and *SOMATIC EMBRYOGENESIS RECEPTOR-like KINASE* (*SERK*) demonstrated that the expression of these genes varied along embryogenesis and are differentially expressed in embryogenic and non-embryogenic cultures in some species (Yang and Zhang, 2010; Klimaszewska et al., 2016). Recently, Alvarez et al. (2018) reported on the characterization of the *WUSCHEL-RELATED HOMEOBOX* gene family in maritime pine and its expression during SE.

In the present work, we develop strategies to overcome the limitations of the induction and maturation phases in maritime pine SE. Specifically, we analyzed the possible maternal influence on the embryogenic capability of megagametophytes from controlled crosses as well as the effect of the temperature and water availability during the SE process on the production of plants. Finally, we studied hormonal and molecular changes occurring during the embryo maturation in two EMs morphotypes to determine the possible mechanism responsible for differences in their maturation capability.

## Materials and Methods

### Plant Material

Initial plant material consisted on immature maritime pine (*Pinus pinaster* Aiton) cones collected in middle-late July from both manually crossed and OP mother trees. Manual crosses were performed in early spring 2011 and 2016, the year before cone collection. After manual pollination, female flowers were covered with plastic bags until cone started to develop. Cones from OP and controlled crosses were used to study the genetic control of SE induction, and OP to study the effect of temperature and water availability on the process. Cones were stored for 3–4 days in the dark at 4°C until used.

### Genetic Control of SE Induction

Genetic control of SE induction was studied using five maritime pine elite clones (B5, B12, B14, B25, B50), from the Soria-Burgos provenance of the species, growing at the Bóveda maritime pine seed orchard (43°52N, 7°93′W; Bóveda, Lugo, Spain). Three independent experiments were performed during the years 2012, 2015, and 2017. Experiments in 2012 and 2017 were performed with cones from OP and controlled crosses and in 2015 were performed with cones from OP mother trees. The number of genotypes and crosses tested varied along the years depending on plant material availability. Data on the cone length was recorded after sampling and the mean number of seeds per cone was recorded after cone sterilization and seed isolation, see below.

The protocol used for SE induction was as described in Cano et al. (2018). Briefly, cones were surface sterilized by immersion in 96% ethanol for 20 min and dried in a laminar flow cabinet, scales removed, seeds isolated, and megagametophytes cultured on a modified mLV medium as described in Humánez et al. (2012) and incubated in the dark at 23°C. Initial embryogenic extrusions were removed from the megagametophyte and subcultured every 15 days onto the same medium for proliferation. The percentage of explants that produced established EMs lines was determined after 90 days. At least 10 petri dishes with 6–10 megagametophytes were prepared for each origin assayed.

### Effect of Temperature and Water Availability on SE

The effect of temperature and water availability on the embryogenic process was studied in three OP mother trees (1007, 1046, and 1058) belonging to the Galician Tree Breeding Program (Conselleria do Medio Rural, Xunta de Galicia, Spain). Cones were collected in middle July 2015 in the seed orchard sited in Cordal da Loba (43°18′N, 07°56′W; Monfero, A Coruña, Spain).

The SE protocol used in this experiment, including somatic embryo induction, proliferation, maturation, germination, plant formation, and acclimatization, was as described in Cano et al. (2018).

To test the effect of temperature on the production of stablished EMs, megagametophytes isolated and cultured as described above were incubated at 18, 23 or 28°C for 40 days, embryogenic extrusions were removed from the megagametophyte and subcultured every 15 days onto the same medium and temperature for proliferation. The percentage of initial megagametophytes that produced established EMs after 90 days of culture initiation was recorded and analyzed. At least 10 petri dishes with 6–10 megagametophytes were prepared for each condition assayed.

To study the effect of temperature conditions on the production of mature somatic embryos, EMs stablished from megagametophytes cultured at 23 or 28°C were proliferated and maturated at 18, 23, and 28°C in a factorial design. For maturation, EMs were disaggregated in liquid medium, recovered on filter paper, transferred to ABA-free prematuration medium for 10 days, and finally transferred to ABA-containing maturation medium (mLV, 80 μM ABA, 6% sucrose, 0.9% Gelrite) as described in Cano et al. (2018). The total number of mature embryos/g FW derived from each line was scored after 120 days. At least six replicates were prepared for each embryogenic line in the maturation assays.

The effect of water availability on the embryogenesis was tested by increasing Gelrite concentration in culture medium from 3 to 6 g/L during the induction and proliferation phases. Water availability, determined after Owens and Wozniak (1991) method, and performed as described in Pereira et al. (2016), was 0.613 vs. 0.578 mg of water for Gelrite concentrations of 3 and 6 g/L, respectively (*p* ≤ 0.05). At least 10 petri dishes with 6–10 megagametophytes were prepared for each condition assayed. After proliferation, EMs were maturated as described above and the total number of mature embryos/g FW derived from each line was scored after 120 days.

### Hormonal Profiles and Expression Patterns of Embryogenesis Genes During the Maturation Phase

ABA and IAA contents were determined in 6 embryogenic lines established from B5 and B14 mother trees. The lines were selected based on their morphology: three were SK and three SM. Samples (200 mg FW) were randomly collected at 5 times during the maturation process: from EMs before maturation, 10 days after their transference to ABA-free-pre-maturation medium, and every 4 weeks after their transference to ABA-containing maturation medium until a total period of 12 weeks. Samples were stored at −80°C. At the beginning of the experiment material consisted of proliferating EMs containing translucid somatic embryos with various long suspensors as described in Humánez et al. (2012); at the end of the fourth week, a mix of EMs and early translucid embryos were found. Between the 4th and the 8th weeks samples contained a mix of PMEs and somatic embryos at the precotyledonary stage as previously described (von Arnold and Hakman, 1988; Tereso et al., 2007). Finally, between the 8–12 weeks samples contained a mix of PEMs, opaque precotyledonary somatic embryos and mature embryos with well-defined cotyledons. Samples from the three lines of each morphotype were homogenized, used to prepare three biological replicates, and endogenous ABA and IAA contents were analyzed by UPLC and MS/MS (Ultra performance liquid chromatography/mass spectrometer) using a Q-Exactive Orbitrap (Thermo Fischer) (VLC/Campus Consortium, IBMCP, Valencia, España). The thoroughly ground tissue was suspended in 80% methanol-1% acetic acid containing internal standards and mixed by shaking during 1 h at 4°C. The extract was kept at −20°C overnight and then centrifuged and the supernatant dried in a vacuum evaporator. The dry residue was dissolved in 1% acetic acid and passed through a reverse phase column (HLB Oasis 30 mg, Waters), as described in Seo et al. (2011). The final residues were dried and dissolved in 5% acetonitrile-1% acetic acid and the hormones were separated by UHPLC with a reverse Accucore C18 column (2.6 μm, 100 mm length; Thermo Fisher Scientific) with a 2–55% acetonitrile gradient containing 0.05% acetic acid, at 400 μL/min over 21 min. The hormones were analyzed with a Q-Exactive mass spectrometer by targeted Selected Ion Monitoring (tSIM; capillary temperature 300°C, S-lens RF level 70, resolution 70.000) and electrospray ionization (spray voltage 3.0 kV, heater temperature 150°C, sheath gas flow rate 40 μL/min, auxiliary gas flow rate 10 μL/min) in negative mode. The ABA and IAA concentrations in the extracts were determined using embedded calibration curves and the Xcalibur 4.0 and TraceFinder 4.1 SP1 programs. The internal standards for quantification of each of the different plant hormones were the deuterium-labeled hormones (purchased from OlChemim Ltd, Olomouc, Czechia).

The expression level of *LEC1* (*PpLEC1*, sp_v2.0-unigene, Sustainpine project, unpublished) and *WOX2* (*PpWOX2*, GenBank accession KY773924.1) genes during the maturation phase was determined by qRT-PCR in 4 embryogenic lines with different morphology (2 SK and 2 SM). Samples were collected at three times along the maturation process (0, 7, and 12 weeks named after 0W, 7W, and 12W, respectively) and stored at −80°C until analyzed. Total RNA was extracted in triplicates using the RNeasy Plant Mini Kit (Qiagen). To eliminate any residual genomic DNA, RNA samples were treated with Recombinant DNase I (RNase-free, Takara Bio, Shiga, Japan) according to the manufacturer’s protocols. The quantity of isolated RNA both before and after DNase treatment was measured using a Nanodrop-TM 2000 (Thermo Fisher Scientific, Waltham, MA, United States). cDNA was synthetized using the PrimeScript RT Reagent Kit (Takara) and relative gene expression was measured by qPCR (StepOne Plus, Applied Biosystems). Three technical replicates were analyzed using *HIS3* (*HISTONE H3*) and *aTUB* (*aTUBULINE)* as internal controls. Reactions were carried out in 20 μL containing 1 μg cDNA, 0.3 μM of each primer and the SYBR green master mix (Takara Bio, Shiga, Japan). The PCR conditions were: initial denaturation at 95°C for 10 min, followed by 40 cycles of 15 s each at 95°C, and 1 min at 51°C. Gene specific primers for *LEC1* and *WOX2* were as described by Hassani (2015) (*LEC1*: 5′-GCTGAAGGCGATCACAGAG-3′ and 5′-AGAACCTGTGATTGAATCCTTG-3′; *WOX2*: 5′-ATCCGGCATCGCTGAATAC-3′ and 5′-TACTTGCCAGGATGCTGAGG-3′); and for *HISTO3* and *aTUB* as described by de Vega-Bartol et al. (2013a) (*HISTO3*: 5′-GCTGAGGCTTACCTTGTG-3′ and 5′-CCAGTTGTATATCCTTAGGCA-3′; and *aTUB:* 5′-ATCTGGAGCCGACTGTCA-3′ and 5′-TGATAAGCTGTTCAGGATGGAA-3′).

Primer efficiencies, calculated according to E = 10^(1/slope)^-1, were similar for different cDNA samples and always greater than 1.81. The relative transcript levels were normalized using *aTUB* and *HIST3* and compared relative to each embryogenic line at time 0W.

### Statistical Analyses

Data recorded in the different experiments were subjected to analysis using the SPSS software (IBM Statistics). Percentage data were arcsin transformed before analysis of variance. When they did not adjust to a normal distribution (Kolmogorov–Smirnoff test), significant differences among the embryogenic response of maritime pine explants were assessed using the Kruskal–Wallis test (more than two groups of data) or the Mann–Whitney *U*-test (two groups of data).

## Results

### Genetic Control of SE Induction

Initially, we present data on cone length and seed production in our OP and controlled crosses. Since availability of maritime pine female cones varied among families and years, statistical analyses were performed separately for experiments performed in 2012, 2015, and 2017. The length of the cones produced by each mother tree did not differ among the sampled years, but depended on the pollination type: OP-derived cones were significantly larger than those derived from manual pollination. Irrespective of the pollination type, the collected cones showed significant differences in size (*P* < 0.001) among the tested mother trees (Table 1). Length of the cones derived from controlled crosses (with at least two different fathers for each mother) did not depend on the paternal genotype.

**Table 1:** Effect of pollination type on the production of maritime pine cones and seeds.

Mother tree	Mean cone length (cm)		Mean number of seeds/cone	
	Open pollinated (Number of years)	Controlled crosses (Number of fathers)	Open pollinated (Number of years)	Controlled crosses (Number of fathers)
B5	15.2 ± 2.5 (3) b	12.1 ± 2.3 (3) bc	141 ± 11 (3) a	31 ± 18 (4) a
B12	19.0 ± 1.7 (2) a	15.1 ± 1.3 (3) a	115 ± 46 (2) ab	15 ± 17 (5) b
B14	13.2 ± 1.1 (3) c	12.5 ± 1.5 (3) b	87 ± 4 (3) bc	24 ± 15 (3) a
B25	11.8 ± 1.5 (2) d	10.2 ± 1.4 (2) d	103 ± 54 (2) ab	13 ± 13 (2) b
B50	12.0 ± 1.5 (3) d	10.6 ± 1.2 (5) cd	61 ± 31 (3) c	19 ± 18 (5) b
Mean	13.1 ± 2.5	12.0 ± 2.2 (5)	93 ± 43	19 ± 17 (7)

*Mean length and mean seed content of female cones harvested in five maritime pine mother trees after open (sampled in 2012, 2015, and 2017) and controlled pollination (sampled in 2012 and 2017). Data, are means ± standard error of at least three cones. Within each column, values followed by the same letter did not differ according to the Mann–Whitney U-test.*

The number of seeds in each cone used in our experiments was not affected by the harvest year and, on average, OP-derived cones contained a higher number of seeds than those derived from controlled pollinations (*P* < 0.001). For both types of cones, we found significant differences (*P* = 0.001) in fertility among the five mother trees employed (Table 1). When analyzed each genotype separately, we found significantly lower seed contents in cones harvested in 2017 from B5 after controlled pollinations (with B12, B14, and B50) and in those derived from open pollination in B50 (40 ± 24 seeds vs. to an average of 85 ± 17 in the two previous years). These results indicate the underlying significant genetic control of these reproductive traits and also suggest that manipulation of female cones affect the fertilization rates in controlled crosses.

Since in the 2015 experiment only cones from three mother trees (B5, B14, and B50) were available, results on SE in maritime pine megagametophytes are analyzed and presented separately for each year (Table 2). Irrespective of the year, the Kruskal–Wallis test revealed significant differences among the embryogenic response of the explants collected from five mother trees (*P* < 0.001 in 2012 and 2015; *P* = 0.023 in 2017). B5 mother tree showed consistently higher percentage of explants that produced EMs than the other four mother trees (on average 35.9 ± 2.5 front to 14.3 ± 2.8). Nevertheless, in the 2017 experiment the response of explants isolated from this mother tree did not significantly differ from that of explants from B12, B14, and B25 genotypes. On the contrary, the embryogenic response of megagametophytes from B14 and B50 mother trees varied with the sampling year (Table 2). Despite of this, the maternal effect on the SE response of megagametophytes was then observed in the three independent experiments.

**Table 2:** Effect of mother tree on the frequency of maritime pine megagametophytes that produced established EMs after 4 months in culture.

Mother tree	Year of the experiment		
	2012	2015	2017
B5	38.0 ± 3.2 a	40.5 ± 3.9 a	30.2 ± 5.0 a
B12	4.0 ± 3.2 c	NT	15.6 ± 5.0 ab
B14	5.1 ± 3.2 c	17.0 ± 3.9 b	21.7 ± 5.1 ab
B25	17.9 ± 3.3 b	NT	12.5 ± 5.0 ab
B50	26.0 ± 3.2 b	22.1 ± 3.9 b	6.3 ± 5.0 b

*Data for each year are means ± standard error of at least 10 replicates with 6–10 explants each. Within each column, values followed by the same letter were not different according to the Mann–Whitney U-test. NT, not tested.*

In SE experiments initiated in 2012 and 2017 we also used explants isolated from cones obtained in controlled crosses among the five different genotypes. Unfortunately, we could not test all the combinations due to failure of several manual crosses (Table 3). On average, the embryogenic potential of these megagametophytes depended on the harvest year (*P* = 0.003), but in both experiments an effect of the maternal genotype on the embryogenic response was observed (*P* < 0.001 in 2012, and *P* = 0.004 in 2017). Again, megagametophytes isolated from cones collected in B5 mother tree showed significantly higher percentages of explants that produced EMs than those isolated from cones collected in the other four mother trees (on average 25.7 ± 2.0 front to 9.8 ± 1.0). Note however that in 2017 the percentage of explants from B5 that produced EMs only differed significantly from that of explants from B50. The effect of the paternal tree was detected only in the 2012 experiment for B14 pollen donor *(P* < 0.001), while results obtained in 2017 showed not significant differences among the five tested fathers (*P* = 0.076). Thus, our results suggest that the paternal genotype has low or not influence in the SE response of maritime pine megagametophytes.

**Table 3:** Effect of mother tree and controlled crosses on the frequency of maritime pine megagametophytes that produced established EMs after 4 months in culture.

Year	Mother	Father					Mean
		B5	B12	B14	B25	B50	
2012	B5	NT	NT	31.1 ± 2.8	NT	35.0 ± 2.7	33.0 ± 1.9
	B12	6.4 ± 2.7	NT	Failed	Failed	NT	
	B14	9.8 ± 2.8	NT	NT	NT	2.9 ± 2.7	
	B25	NT	Failed	NT	NT	6.7 ± 3.8	
	B50	7.5 ± 3.8	NT	NT	10.5 ± 2.7	NT	9.0 ± 2.3
2017	B5	NT	27.6 ± 6.1	5.3 ± 5.1	NT	14.3 ± 6.4	15.7 ± 3.5 ab
	B12	27.8 ± 4.4	NT	12.5 ± 4.2	17.2 ± 4.2	NT	19.2 ± 2.5 a
	B14	6.1 ± 5.1	16.1 ± 4.2	NT	NT	6.3 ± 4.2	9.5 ± 2.6 b
	B25	NT	12.5 ± 6.0	NT	NT	10.4 ± 4.2	11.5 ± 3.7 ab
	B50	NT	7.8 ± 4.2	0.0 ± 0.0	0.0 ± 0.0	NT	2.6 ± 3.0 c

*Data for each year are means ± standard error of 10 replicates with 6–10 explants each. For data from the 2017 experiment, values followed by the same letter were not different according to the Mann–Whitney U-test. NT, not tested.*

### Effect of Temperature and Water Availability on SE

The culture incubation temperature significantly affected the induction and establishment of EMs in maritime pine megagametophytes from three mother trees (Table 4). Thus, no EMs were established when cultures were initiated at 18°C, although callus extrusion could be initially observed in 2.1% of the explants (data not shown). The percentage of explants that produced EMs depended on the family but was not affected when temperature increased from the 23°C in standard conditions up to 28°C (*P* = 0.280) or when Gelrite concentration of the induction medium was increased from 3 to 6 g/L (*P* = 0.668) (Table 4).

Embryogenic masses induced and established on medium with 3 g/L Gelrite at 23° or 28°C, were further proliferated for 45 days at 18°, 23° or 28°C. After this period, EMs from four lines belonging to each family (two induced at 23° and two at 28°C) and proliferated at different temperatures, were transferred to maturation medium, and incubated at 18°, 23°, or 28°C. This factorial design allowed us to study somatic embryo production at 18 different temperature combinations. When averaged throughout families and treatments, the SE induction temperature significantly affected the number of mature embryos obtained from these EMs (*P* < 0.001); thus, embryogenic lines derived from EMs induced at 28°C produced more mature embryos (11.6 ± 2.3 vs. 9.0 ± 2.7 embryos/g FW for 1007 mother tree, 90.5 ± 15.0 vs. 20.8 ± 17.8 embryos/g FW for 1046 mother tree, and 31.9 ± 3.8 front to 15.1 ± 3.3 embryos/g FW for 1058) than those derived from EMs induced at standard conditions.

**Table 4:** Effect of incubation temperature and mother tree on the frequency (%) of maritime pine megagametophytes that produced established EMs after 90 days in culture.

Mother tree	Incubation temperature		
	18°C	23°C	28°C
1007	0.0 ± 0.0 b	9.8 ± 2.1 a	4.3 ± 2.1 a
1046	0.0 ± 0.0 b	4.8 ± 2.0 a	4.3 ± 1.9 a
1058	0.0 ± 0.0 b	10.9 ± 5.5 a	20.8 ± 7.2 a

*Data are means ± standard error of 20 replicates with 6–10 explants each. For each mother tree, values followed by the same letter were not different according to the Mann–Whitney U-test.*

**FIGURE 1:**
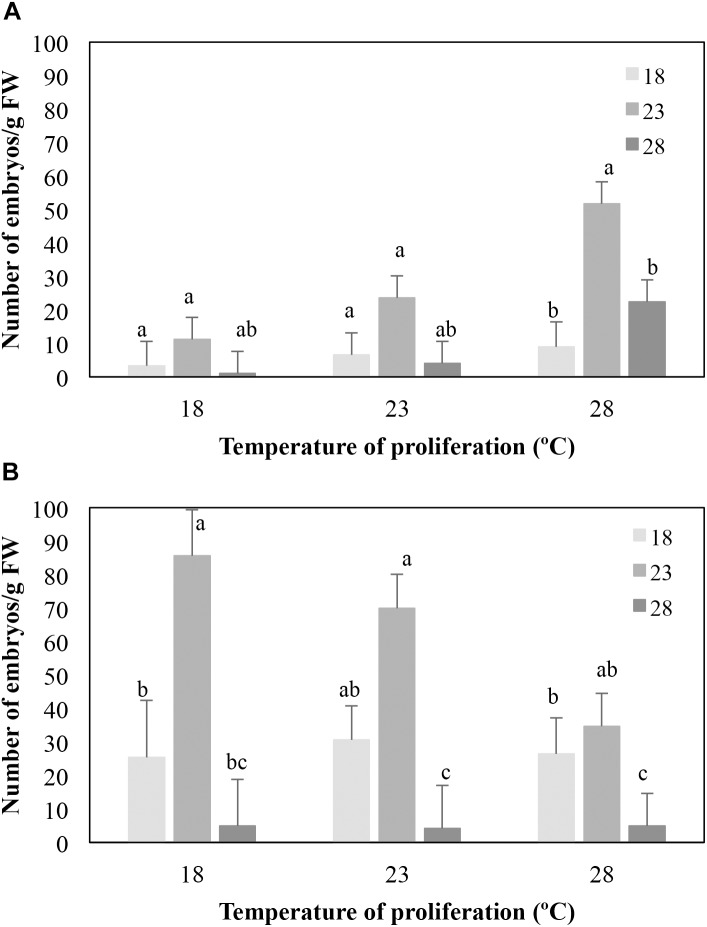
Effect of the temperature of maturation on the number of mature embryos/g FW produced by EMs from four lines derived from maritime pine megagametophytes (Family 1058) induced at 23° **(A)** or 28° **(B)**, and proliferated at three different temperatures. Data are means ± standard error from six replicates for each line. For each set of data, values followed by the same letter were not significantly different according to the Mann–Whitney *U*-test.

Variation found in the number of embryos/g FW obtained from EMs cultivated at different temperatures during the proliferation and maturation phases was only analyzed for the family 1058. The other two families were excluded from the analyses since some treatment combinations, mainly those from EMs cultured at 18°C, were lost. Differences in both proliferation (*P* = 0.006) and maturation (*P* = 0.001) temperatures significantly affected embryo differentiation in EMs induced at 23°C (Figure 1A). Thus, when averaged throughout the temperatures of maturation, the mean number of embryos was higher when EMs proliferated at 28°C than when proliferation was performed at 18°C (27.7 ± 4.0 vs. 5.2 ± 3.9 embryos/g FW, respectively), while no significant differences were observed when compared these two treatments to embryo differentiation of EMs proliferated at 23°C (mean 11.4 ± 3.8 embryos/g FW). On average, higher embryo maturation rates were obtained in EMs maintained at 23°C during maturation (28.8 ± 3.8 embryos/g FW front to 6.3 ± 4.1 and 9.1 ± 3.8 embryos/g FW from EMs maturated at 18 and 28°C, respectively, Figure 1A).

The proliferation temperature did not affect the number of embryos/g FW produced (*P* = 0.286) from EMs induced at 28°C (Figure 1B), but embryo differentiation depended on the temperature of maturation (*P* < 0.001). As described for EMs lines induced at 23°C, higher embryo production rates were observed in EMs maturated at 23°C (58.6 ± 6.5 embryos/g FW front to 28.1 ± 7.0 and 4.7 ± 7.0 embryo/g FW obtained from EMs maturated at 18° and 28°C, respectively, Figure 1B).

As stated above, a complete data set of 18 temperature combinations from families 1007 and 1046 could not be analyzed. Nevertheless, it was also found that EMs induced at 23°C from family 1007 produced on average more embryos/g FW when maturated at 23°C (13.2 ± 3.7 vs. 4.7 ± 3.7 at 28°C); lines from family 1046 showed the same trend (30.2 ± 6.3 embryos/g FW at 23°C vs. 14.0 ± 6.5 at 18°C). Similar results were observed for EMs induced at 28°C, since lines from family 1007 produced on average 18.6 ± 3.7 embryos/g FW when maturated at 23°C vs. a mean of 12.6 ± 4.0 and of 3.9 ± 3.7 when maturated at 18° and 28°C, respectively. EMs from family 1046 produced an average of 5.9 ± 8.9; 126.6 ± 26.2 and 93.6 ± 33.8 embryos/g FW when maturation was carried out at 18, 23°, and 28°C, respectively.

**FIGURE 2:**
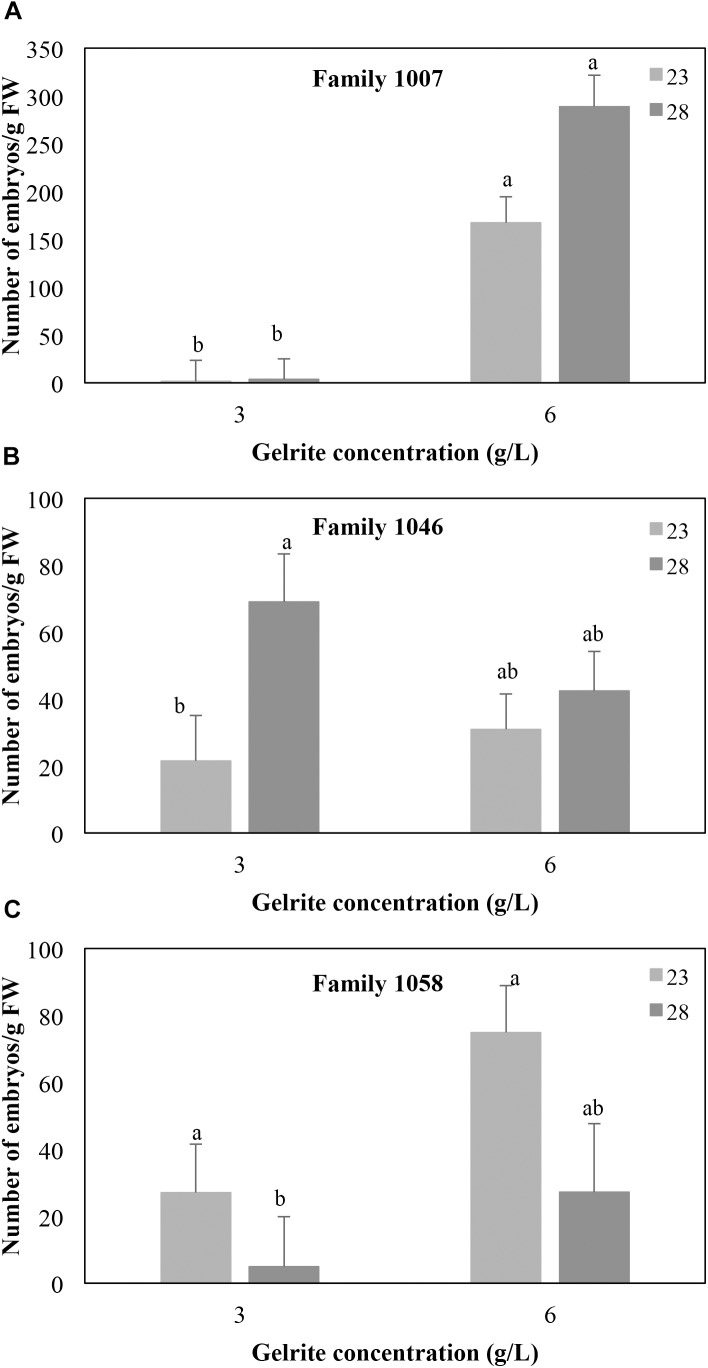
Effect of Gelrite concentration (3 or 6 g/L) and temperature during the induction and proliferation phases on the number of mature SE/g FW from megagametophyte-derived EMs from three maritime pine families 1007 **(A)**, 1046 **(B),** and 1058 **(C)**. Data are means ± standard error of at least six replicates. Within each family, values followed by the same letter did not significantly differ according to the Mann–Whitney *U*-test.

Besides the effect of the incubation temperatures above described, the number of mature embryos produced by maritime pine EMs also depended on the Gelrite concentration of the media, although the response varied among the three families tested. Irrespective of the temperature, EMs from genotype 1007 cultured on medium with 6 g/L Gelrite, significantly increased the number of mature embryos/g FW produced (217.3 ± 22.2 vs. to 2.5 ± 16.5 for 6 g/L and 3 g/L Gelrite, respectively; *P* < 0.001) (Figure 2A). For this mother tree, embryo production did not significantly vary (*P* = 0.728) when the embryogenesis process was performed at 28°C or at standard temperature conditions (23°C). However, increasing Gelrite concentration in media did not affect embryo differentiation in EMs from family 1046 (*P* = 0.316) but affected the response of explants to temperature (Figure 2B). In this respect, the number of embryos g/FW produced by these EMs at 28°C was higher than at 23°C (69.0 ± 14.4 vs. 21.6 ± 13.6 embryos/g FW, respectively) but only in cultures supplemented with 3 g/L Gelrite, while response of EMs cultured in medium with 6 g/L was not affected by temperature (mean 36.7 ± 7.9 embryos/g FW). For EMs derived from genotype 1058 (Figure 2C), embryo differentiation rates depended on both temperature (*P* = 0.002) and Gelrite concentration (*P* = 0.017), although temperature variation only affected significantly (*P* = 0.015) cultures in medium supplemented with 3 g/L Gelrite for which embryo maturation rates were higher for EMs incubated at 23°C (27.0 ± 14.4 embryos/g FW) than for those cultured at 28°C (4.9 ± 14.9 embryos/g FW).

**FIGURE 3:**
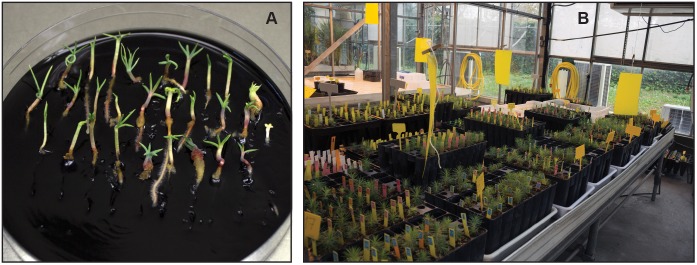
Germination of maritime pine SE on activated charcoal-containing medium **(A)**. Plants derived from SE growing in the greenhouse **(B)**.

Embryos obtained in this experiment were germinated (Figure 3A) at standard conditions (23°) as described in Cano et al. (2018). Germination percentages differed among families (56, 49, and 83% for families 1007, 1046, and 1058, respectively). When averaged throughout families, higher germination rates were detected for mature embryos produced by EMs proliferated at 28°C (on average 62%), than for those produced by EMs proliferated at 18°C (41%) or at 23°C (52%). We also found higher germination rates in embryos derived from EMs maturated at 23° (78%) than from EMs maturated at 18° (50%) or 28°C (34%). A total of 930 plants were obtained in this experiment (Figure 3B), although again this result depended on the family: 207 were derived from 1007 family, 243 from 1046 family, and 480 from 1058 family (93 from EMs induced at 23°C and 387 from EMs induced at 28°C).

According to these results, it seems that optimal temperature conditions for embryogenesis in maritime pine megagametophytes are different for each embryogenic phase. When induction was performed at 28°C instead that at 23°C, the percentage of responding explants was not affected, but the number of mature embryos produced by the obtained EMs was significantly increased. This temperature resulted also more adequate during the proliferation phase, while maturation should be performed at 23°C to maximize the number of embryos and plants produced. The yield of the maritime pine SE protocol could also be increased by reducing water availability in induction and proliferation media by adding 6 g/L Gelrite.

### Hormonal Profiles and Expression Patterns of Embryogenesis Genes During the Maturation Phase

**FIGURE 4:**
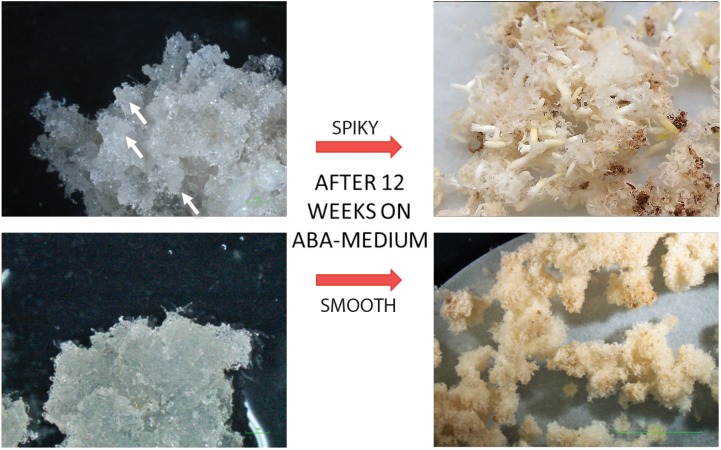
Morphology of spiky (SK, **up**) and smooth (SM, **down**) maritime pine embryogenic lines and their appearance before **(left)** and after **(right)** 12 weeks in maturation medium.

Embryogenic masses lines with different morphology were used to characterize the maturation phase. SK embryogenic lines showed a higher maturation capacity than SM lines after 12 weeks on maturation medium (on average of 77.8 vs. 2.1 mature embryos/g FW, Figure 4).

**FIGURE 5:**
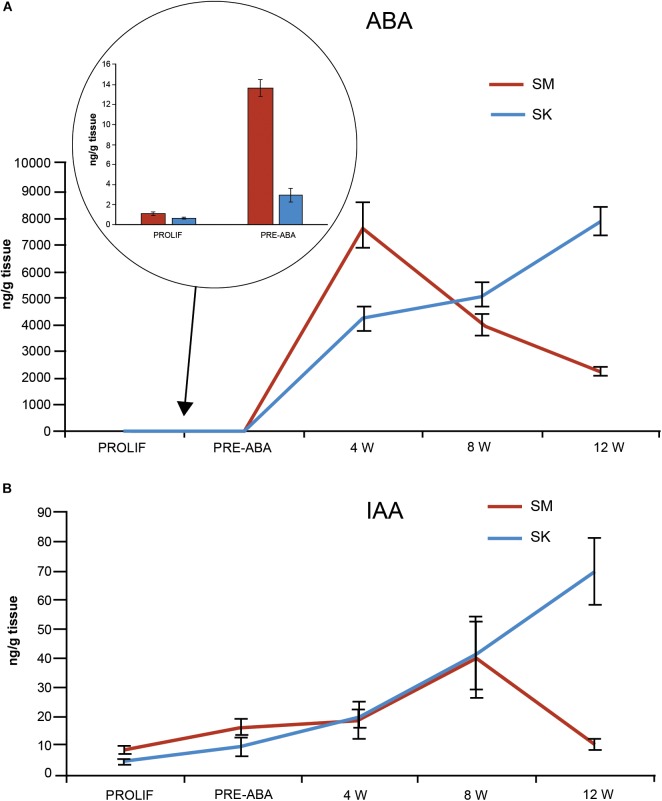
ABA **(A)** and IAA **(B)** content (ng/g FW) in EMs with SK (blue) and SM (red) morphology at different times before and during maturation. For maturation, EMs were first transferred for 10 days to ABA-free medium (data in the circle) and then to ABA containing medium. Data are means ± standard error of three biological replicates.

Endogenous contents of ABA and IAA, two hormones related to zygotic embryo maturation (von Aderkas et al., 2001), were determined at the end of proliferation phase and at four points during maturation (Figure 5). ABA levels on proliferation medium (before maturation started) did not differ between both morphotypes (average of 0.70 ± 0.09 and 1.03 ± 0.014 ng ABA/g FW for SK and SM, respectively; Figure 5A). After transference to ABA-free maturation medium and then to ABA-maturation medium, a steady significant increase in endogenous ABA content was observed in both morphotypes, that picked after 4 weeks of the maturation process in SM lines (up to 7755.9 ± 830.6 ng ABA/g FW), after which drastically decreased. In contrast, ABA content in SK lines progressively increased throughout the whole maturation process up to 5138.6 ± 438.4 ng ABA/g FW (Figure 5A). Endogenous IAA content was higher in SM than in SK lines in proliferation medium and especially after 10 days of transference to ABA-free maturation medium (16.2 ± 2.5 vs. 9.5 ± 3.1 ng IAA/g FW for SM and SK lines, respectively; Figure 5B), it was similar in both morphotypes at 4 and 8 weeks and then decreased in SM lines, while in SK lines picked at 12 weeks (69.7 ± 9.5 ng IAA/g FW; Figure 5B).

**FIGURE 6:**
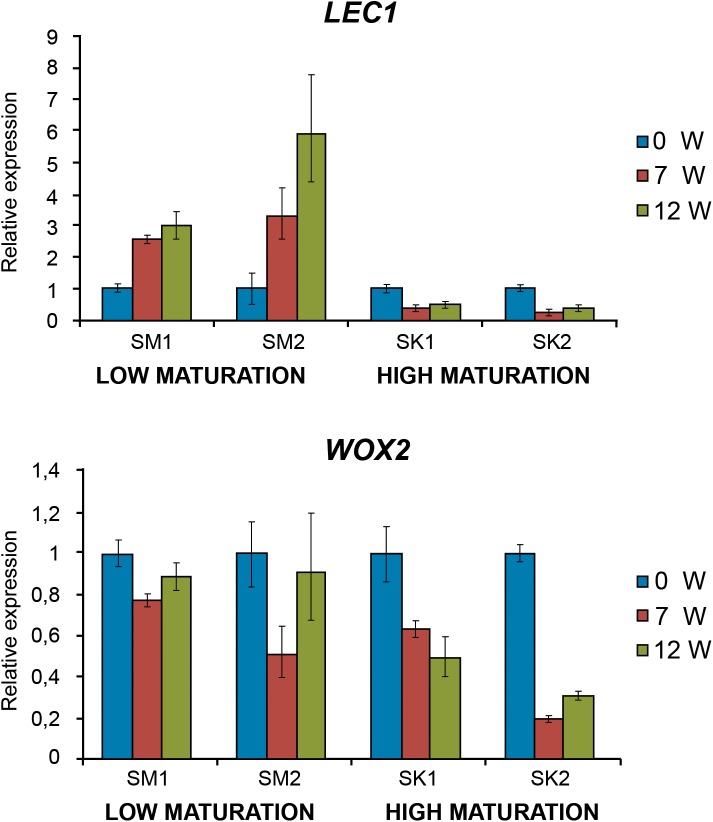
*LEC1* **(A)** and *WOX2* **(B)** fold-relative gene expression along maturation in EMs with low (SM) and high (SK) maturation capacity. Data are means ± standard error of three biological replicates.

Two different embryogenic lines for each morphotype were also employed to investigate the expression profiles of genes coding for LEC1 and WOX2 transcription factors involved in the embryogenic process. Relative expression of *LEC1* progressively decreased along with maturation time in SK lines that showed a high maturation capacity (Figure 6), while *LEC1* transcripts increased up to 6-fold in lines with SM with low maturation capacity. Similarly, *WOX2* expression significantly decreased in SK lines while remaining high in SM lines (Figure 6).

## Discussion

### Effect of Genotype on Maritime Pine SE

Maritime pine SE has been achieved using explants collected in trees from different provenances, including selected genotypes growing in a continental region of Spain (Humánez et al., 2012). The established protocol for this plant material includes harvesting cones at the end of July, to isolate megagametophytes containing a zygotic embryo at the precotiledonary stage, and their cultivation on a modified Litvay’s medium(Litvay et al., 1985). SE was then induced on 58.6% of the explants, although a significant mother tree effect was observed. To further study this issue, here we first tested the effect of genotype on the efficiency of the protocol using cones derived from OP and from controlled crosses, collected in five selected mother trees growing in this location. Genetic differences among the five mother trees accounted for significant variation in cone length and fertility (Table 1), and also for the percentage of SE induction on the half-sib families of megagametophytes, excised from open-pollinated cones (Table 2). The significant maternal effect was also observed when averaged for each genotype data from full-sib families (Table 3), while paternal effect was mostly undetectable. Furthermore, explants excised from cones collected in genotype B5 showed consistently higher induction rates, but no significant increases were observed for controlled crosses in which this genotype was used as pollen donor.

Our results agree with previous references, since conifer SE has been probed to depend largely on genotype, and the genetic control of SE initiation has been studied in several *Pinus* species. Niskanen et al. (2004) reported a strong maternal effect in *P. sylvestris* SE initiation and embryo production, while only a less significant paternal effect was detected for SE initiation. Authors did not find significant specific combining ability, neither a correlation for SE initiation ability when the same genotype was used as mother or father. This lack of correlation between female and male SE initiation values was also reported by MacKay et al. (2006) for *Pinus taeda*. For this plant species, general combining ability was found to account for a large proportion of variance at SE initiation, although maternal effects may mask the additive genetic effects detected. Pereira et al. (2016) referred initiation rates of embryonal masses significantly different among five tested *Pinus halepensis* mother trees. Finally, Miguel et al. (2004) found significant differences for SE initiation among 20 *P. pinaster* half-sib families (results ranged from 2.0 to 35.8%), thus indicating the existence of a maternal genetic effect for SE initiation in plant material from Portugal. Both the genotype and the developmental or physiological stage of the mother tree, as well as the inherited maternal alleles of the zygotic embryo can explain the maternal effect. Also, variations on the composition of the fluid in the corrosion cavity, that include nutrients and hormones, provided by the megagametophyte to nourish the zygotic embryo after fertilization (Carman et al., 2005), may account for differences in their embryogenic capacity. Differences on the physiological stage of the mother tree may also help to explain variation on the response of megagametophytes collected in different years from the same genotype. In this regard, Kvaalen and Johnsen (2008) reported that the induction of embryogenesis in *Picea abies* was clearly influenced by the temperature during zygotic embryogenesis. In addition, Pullman et al. (2015) reported beneficial effect of the redox environment of seed on the initiation of embryogenic tissue in loblolly pine.

Irrespective of the genotype, open-pollinated cones were longer and contained significantly higher number of seeds than those derived from controlled pollination. However, when results obtained in the 2013 and 2017 experiments were compared, the origin of cone did not affect the capability of the megagametophytes for induction of SE. Note, however, that explants excised from open–pollinated cones of genotype B50 showed significantly (*P* = 0.001) higher induction rates (17.2 ± 3.0) than those isolated from cones derived from manual pollination (6.3 ± 2.0). It is common in pines that seeds of open-pollinated families respond to SE initiation better than seeds from cross-pollinated families (Percy et al., 2000; Miguel et al., 2004), but this was not the general trend in our experiment. A maritime pine molecular marker study reported outcrossing ratios in OP progeny to be close to one (Gaspar et al., 2009), then possible differences in SE induction of OP-derived megagametophytes as compared to those derived from controlled crosses could not be explained by the presence of self-pollination derived explants in OP families. Genetic differences, however, may account as a consequence of the distinct pool of fathers which contribute to form both progenies. The necessary manipulation and covering of female cones can also affect the success of controlled pollination and the conditions during the initial development of zygotic embryos, then producing a progeny with altered characteristics.

### Effect of Temperature and Water Availability on SE

Our second experiment was performed to study the effect of temperature and medium water availability on *Pinus pinaster* SE, which is generally carried out at 23°C and induced using media gelified with 3–4 g/L Gelrite (Lelu-Walter et al., 2006; Humánez et al., 2012; Cano et al., 2018). No embryogenic lines were obtained from megagametophytes cultured at 18°C (Table 4), while increasing temperature up to 28°C resulted on initiation rates similar to those observed in standard conditions, but significantly increased the number of somatic embryos produced. Furthermore, a warmer temperature during the proliferation phase significantly increased embryo production of lines induced at 23°C, while those induced at 28°C remained unaffected. Finally, higher embryo differentiation rates were observed in cultures incubated at 23°C during maturation, irrespective of the temperature of induction (Figure 1). In contrast, García-Mendiguren et al. (2015), reported that *Pinus radiata* explants showed higher initiation rates when induction was performed at 18°C instead of 23°C, while increasing temperature up to 28°C significantly decreased SE induction. However, lines cultured at 28°C showed significantly higher number of somatic embryos than those cultured at either 18° or 23°C, which is in agreement with our results for maritime pine. The effect of temperature and Gelrite concentration on SE was also tested in *Pinus halepensis*: higher rates of induction and proliferation were obtained in cultures incubated at 23°C as compared to 18° or 28°C and in medium supplemented with 4 g/L Gelrite as compared to 2 or 3 g/L (Pereira et al., 2016). However, these factors did not affect the final number of embryos/g obtained from each cell line. Environmental conditions affected similarly SE in *Picea abies*, since lowering the temperature from 23 to 18°C during proliferation significantly reduced the growth of the induced EMs, while cultures kept at 28°C tended to produce a higher number of mature embryos than cultures kept at 18 and 23°C (Kvaalen and Johnsen, 2008). In this respect, Bonga et al. (2010) pointed out that lowering or increasing temperatures may improve the initiation and proliferation stages as they act as a mild stress that favors cell reprogramming, whereas other authors (Li and Wolyn, 1996), suggested that increasing temperatures during proliferation phase may increase the production of storage reserves in SE.

Recently, reported improvements in SE protocols for different conifer species, including *Pinus pinaster*, were reviewed by Klimaszewska et al. (2016). Significant increases on EMs induction and growth have been derived mainly from variations in media formulation (sugars, vitamins, organic acids, growth regulators), while embryo maturation has been improved, among other strategies, by adding activated charcoal or compounds that modify medium water potential (carbohydrates, polyethylene glycol) or water availability (gelling agents). Increasing gelling agent concentrations in the culture medium reduces its matric potential and thereby decreases water potential and limit ion uptake, which reduces tissue water availability (George, 1993). In our experiments, increasing Gelrite concentration did not affect somatic embryo induction rates, but significantly affected their differentiation, being the response depending on the genotype of the mother tree and influenced by temperature (Figure 2). In most of the species studied (Morel et al., 2014a; García-Mendiguren et al., 2015; Pereira et al., 2016; Klimaszewska et al., 2016), increasing agar concentration also favored embryo maturation. Interestingly, lower levels of water availability also resulted in higher initiation rates in *P. taeda* (Pullman and Johnson, 2002), and *P. radiata* (García-Mendiguren et al., 2015).

From our results, it seems possible to improve somatic embryo production of maritime pine megagametophytes by adjusting optimal temperature throughout the process: 28°C during induction and proliferation, and 23°C during the maturation phase.

### Changes in Endogenous Hormonal Content and in the Expression of *LEC*1 and *WOX*2 Genes Revealed Correlation Between EMs Morphotypes and Maturation Competence in Maritime Pine

Cellular composition of conifer EMs is highly heterogeneous and changes over culture time (Klimaszewska et al., 2016). In maritime pine, it was demonstrated that spatial separation (in different Petri dishes) of EM sublines (from the same genotype) could induce random, reversible changes in SE maturation yield as a consequence of environmentally induced modifications of gene expression. This different maturation capacity correlated with different morphology (Breton et al., 2006), as was observed in our experiments (Figure 4). In an attempt to identify the underlying causes of this phenomenon, studies have been conducted to determine endogenous hormone content and modifications on the expression of embryogenesis-related genes.

In almost all conifer species, somatic embryo maturation requires the transference of the proliferating cultures of early somatic embryos (after a pretreatment or without it) onto a medium with ABA and a low water potential provided by increasing sugar and/or gelling agent concentration (reviewed in Klimaszewska et al., 2016; Lelu-Walter et al., 2016). Although in some conifer species somatic embryos maturation can be achieved without ABA, those derived from ABA-supplemented medium displayed a coordinated growth, better morphology and the concomitant accumulation of lipids and storage proteins, while these features were lacking in embryos developed in the absence of ABA (Klimaszewska et al., 2016). Comparative studies on zygotic and somatic embryos have been performed in several conifer species regarding gene expression (Lara-Chavez et al., 2012; Morel et al., 2014b; Bravo et al., 2017), hormonal content (von Aderkas et al., 2001) and storage proteins(Tereso et al., 2007).

In the present work, ABA profiles during maritime pine somatic embryo maturation differed between SK and SM lines (Figure 5A). On SK lines ABA have a steady increase during the 12 weeks of the process, as has already been reported for *Picea abies* (von Aderkas et al., 2001). In contrast, SM lines significantly increased ABA concentration as soon as after 10 days on ABA-free-prematuration medium, picked at 4 weeks on ABA-containing maturation medium and drastically decreased until the end of the experiment (Figure 5A). Morel et al. (2014a) analyzed endogenous ABA content, for a period of 4 weeks, in embryogenic lines maturated under favorable (9 g/L Gelrite) and unfavorable conditions (4 g/L Gelrite). Although our experimental conditions are not comparable to those of Morel et al. (2014a), note that in both experiments endogenous ABA significantly increased after 4 weeks of maturation. We suggest that ABA high content at the initial maturation stages along with a drastic decrease when the precotyledonary embryos are formed (8 W) may account for the low maturation rates of SM lines. Storage protein accumulation at the middle-end SE maturation, induced by ABA, has been reported as a potential indicator of good quality somatic embryos in maritime pine(Tereso et al., 2007).

Although factors controlling ABA changes have not been investigated in this work, it is well known that9-*cis*-epoxycarotenoid dioxygenases (NCED) and CYP707A cytochrome P450 monooxygenases control biosynthesis and catabolism of the hormone, respectively (Liao et al., 2018). Transcriptional regulation of both genes may be regulated by endogenous and exogenous environmental conditions (Liao et al., 2018), and also, by epigenetic mechanisms activated specially during drought stress (Virlouet and Fromm, 2015), embryo development (Pérez et al., 2015), and seed dormancy and germination (Nonogaki, 2014; Boccaccini et al., 2016).

A large body of experimental observations exists on the central role of endogenous IAA in the regulation of embryo development (Zhou et al., 2017). In conifers, induction of EMs requires de presence of the synthetic auxin 2,4-D, but it appears that the decrease in IAA level at the start of maturation may be the most important step for the subsequent development of embryos (Abrahamsson et al., 2011; Vondráková et al., 2011; Zhou et al., 2017). In fact, the auxin transport inhibitor 1-*N*-naphtylphthalamic acid (NPA), improved maturation in *Picea abies*. In addition, an increase in IAA levels at the late stages of SE has also been reported in developing zygotic embryos of conifers (Sandberg and Ernstsen, 1987; Souter and Lindsey, 2000). In this work, SM lines had higher IAA content at the start of the maturation phase and low content at the end of the process, which may account for the low maturation capability of these lines. On the contrary, SK lines with less content of IAA at the initial, and higher at the end of the maturation phase, produced higher rates of well- formed cotyledonary somatic embryos (Figure 5B). The molecular mechanisms that regulate auxin biosynthesis at transcriptional and protein level are virtually unknown but the integration of environmental signals to regulate auxin biosynthesis seems to be a common mechanism to adapt plant growth behavior to the ever-changing surroundings (Ruiz et al., 2012; Zhao, 2018). In this respect, de Vega-Bartol et al. (2013b) studied transcriptome changes during zygotic embryo maturation in maritime pine and found a strong down regulation of a putative *AUXIN RESPONSE FACTOR (ARF16)* during the first days of embryo formation, showing the relevance of auxin response mechanisms in the early embryogenesis stage, and an increase of *ARF16* transcripts from early cotyledonary to mature embryos.

Taken all this in account, our results suggest that environmental conditions occurring during tissue culture manipulation may induce stress conditions in the EMs of SM morphotypes that altered ABA and IAA contents that in turn may be partially responsible for its low maturation capability.

Previous studies about the molecular mechanisms regulating the early phase of SE in gymnosperms have revealed genes with expression patterns regulated differentially between embryogenic and non- embryogenic tissue (Stasolla et al., 2004; Palovaara et al., 2010). Recently, epigenetic mechanisms have emerged as being critical in control of both somatic and zygotic embryogenesis by ultimately determining gene expression patterns (Lelu-Walter et al., 2016). The switch from EMs proliferation to SE development and maturation has also recently been investigated by both transcriptome and proteome profiling (Morel et al., 2014a; Plomion et al., 2016; Trontin et al., 2016). Physiological and metabolic changes occurring from EMs to mature somatic embryos are attributed to the activity of regulatory genes such as *SERK*, *LEC1* and *WOX2* (Mahdavi-Darvari et al., 2015).

*LEC1* gene codes for a HAP3-subunit of the CCAAT-box binding transcription factor CBF, known as NF-Y (NUCLEAR FACTOR-Y). LEC1 acts as a master regulator for embryogenesis and controls the activation of further members of the LEC gene family (Rupps et al., 2016). In Arabidopsis, the ectopic expression of *LEC1* causes the induction of SE in the absence of exogenous auxin (Braybrook and Harada, 2008), then these authors propose that LEC Transcription Factors, via *LEC2*, activate auxin biosynthetic enzymes, and that an increase in endogenous auxin levels serves to induce SE; *LEC1* expression, however, ceases prior to final embryo maturation (Perruc et al., 2007). Similarly, *LEC1* gene was also mainly expressed during early embryogenesis in *Larix decidua* (Rupps et al., 2016), which is in accordance with our result in SK morphotypes. Downregulation of *LEC1* gene at the end of the maturation phase could implicate changes in endogenous hormone content; in this respect Braybrook and Harada (2008) suggest that a potential thread between the roles of LEC TFs in the maturation phase and SE might be their involvement in controlling the ABA to GA balance, being higher during the maturation process and low before germination.

*WOX2*, the *WUS* homolog gene, has been proposed as an embryogenic potential marker for some *Picea* and *Larix* species, since it is not present in non-embryogenic lines (Palovaara and Hakman, 2008; Park et al., 2010). In contrast, *WOX2* expression was detected in both embryogenic and non-embryogenic lines in *P. radiata*, although transcript levels were lower in the latter (Bravo et al., 2017). Maritime pine *WOX* gene family has been studied recently (Alvarez et al., 2018) and *WOX2* expression has been shown to be highest at early embryogenic stages and decreased along the maturation process being lower in mature somatic embryos which agree with our *WOX2* expression profile sampled at 0, 7, and 12 weeks in SK lines. Similar results were obtained in other conifers as *Pinus sylvestris* (Merino et al., 2018), *Picea* (Palovaara and Hakman, 2008; Klimaszewska et al., 2011), *Larix decidua* (Rupps et al., 2016), *Pinus contorta* (Park et al., 2010), and Chinese fir (Zhou et al., 2017).

Results presented here show a decrease in the expression of both *WOX2* and *LEC1* genes along the maturation phase but only on *P. pinaster* SK lines with maturation capacity (Figure 6). Interestingly, Merino et al. (2018) did not find significant differences in *WOX2* expression patterns of two scots pine embryogenic lines that differed in their ability to produce normal embryos. Those differences were, on the contrary, detected for the patterns of transcripts accumulation on a *LEC1*-type HAP3 gene. Although in maritime pine differential *WOX2* expression between embryogenic and non-embryogenic cells has not been reported, in our work *WOX2* and *LEC1* expression was detected in both EM morphotypes, suggesting their embryogenic identity (Figure 6). Then, differences in maturation capability may be due to other factors implicated in the downregulation of both genes along the maturation process, such as other hormone contents as has been reported in a recent study in Norway spruce (Vondrakova et al., 2018), or may be regulated by another genes related with embryo development.

## Conclusion

Our results provide new insides for the use of SE as an important tool in maritime pine breeding. Thus, selected mother genotypes with high embryogenic capability may be used in controlled crossed to introduce paternal desirable traits in the progenies. The optimized protocol to produce maritime pine plants with the desirable traits should include the culture of megagametophytes at 28°C for EMs induction and further proliferation, and maturation at 23°C.

The characterization of the possible epigenetic marks in the obtained plants caused by temperatures applied during the embryogenic process as well as the genetic stability of the obtained plants will be the objectives of further investigation.

Our results also demonstrated that maturation capability of maritime pine EMs are positively associated with low levels of ABA and IAA during the first 2–3 weeks on maturation medium and an increased level of both phytohormones at the end of the embryo development. We also demonstrated that down regulation of *LEC1* and *WOX2* genes is required for somatic embryo maturation in maritime pine. Although for practical purposes morphological selection of embryogenic lines seems to be the simplest methodology to facilitate the formation of mature somatic embryos, our results regarding *LEC1* and *WOX2* differential gene expression between morphotypes are of interest to enlighten the process underlying maritime pine somatic embryo maturation.

## Author Contributions

IA, JS, and ES conceived and designed the experiments. IA, ES, MM, and IZ performed the experiments. IZ and ES analyzed the data. FL designed and performed the controlled crosses and collected cones. IA, ES, and JS wrote the manuscript.

## Conflict of Interest Statement

The authors declare that the research was conducted in the absence of any commercial or financial relationships that could be construed as a potential conflict of interest.

## Abbreviations

EMs: embryogenic masses
FW: fresh weight
OP: open pollinated
SE: somatic embryogenesis
SK: spiky morphotype
SM: smooth morphotype.
